# Generation and external validation of a tumor-derived 5-gene prognostic signature for recurrence of lymph node-negative, invasive colorectal carcinoma

**DOI:** 10.1002/cncr.27628

**Published:** 2012-05-17

**Authors:** Peter F Lenehan, Lisa A Boardman, Douglas Riegert-Johnson, Giovanni De Petris, David W Fry, Jeanne Ohrnberger, Eugene R Heyman, Brigitte Gerard, Arpit A Almal, William P Worzel

**Affiliations:** 1 Everist Genomics, Inc.Ann Arbor, Michigan; 2 Division of Gastroenterology and Hepatology, Mayo Clinic RochesterRochester, Minnesota; 3 Division of Gastroenterology and Hepatology, Mayo Clinic JacksonvilleJacksonville, Florida; 4 Department of Laboratory Medicine and Pathology, Mayo Clinic ScottsdaleScottsdale, Arizona; 5 Independent Statistical ConsultantMontgomery Village, Maryland

**Keywords:** colorectal cancer, gene expression signatures, machine learning, recurrence, reverse transcriptase-polymerase chain reaction, prognosis, validation studies, sensitivity and specificity, colonic polyps

## Abstract

**BACKGROUND:** One in 4 patients with lymph node-negative, invasive colorectal carcinoma (CRC) develops recurrent disease after undergoing curative surgery, and most die of advanced disease. Predicting which patients will develop a recurrence is a significantly growing, unmet medical need. **METHODS:** Archival formalin-fixed, paraffin-embedded (FFPE) primary adenocarcinoma tissues obtained at surgery were retrieved from 74 patients with CRC (15 with stage I disease and 59 with stage II disease) for Training/Test Sets. In addition, FFPE tissues were retrieved from 49 patients with stage I CRC and 215 patients with stage II colon cancer for an External Validation (EV) Set (n = 264) from 18 hospitals in 4 countries. No patients had received neoadjuvant/adjuvant therapy. Proprietary genetic programming analysis of expression profiles for 225 prespecified tumor genes was used to create a 36-month recurrence risk signature. **RESULTS:** Using reverse transcriptase-polymerase chain reaction, a 5-gene rule correctly classified 62 of 92 recurrent patients and 87 of 172 nonrecurrent patients in the EV Set (sensitivity, 0.67; specificity, 0.51). “High-risk” patients had a greater probability of 36-month recurrence (42%) than “low-risk” patients (26%; hazard ratio, 1.80; 95% confidence interval, 1.19-2.71; *P* = .007; Cox regression) independent of T-classification, the number of lymph nodes examined, histologic grade/subtype, anatomic location, age, sex, or race. The rule outperformed (*P* = .021) current National Comprehensive Cancer Network Guidelines (hazard ratio, 0.897). The same rule also differentiated the risk of recurrence (hazard ratio, 1.63; *P* = .031) in a subset of patients from the EV Set who had stage I/II colon cancer only (n = 251). **CONCLUSIONS:** To the authors' knowledge, the 5-gene rule (OncoDefender-CRC) is the first molecular prognostic that has been validated in both stage I CRC and stage II colon cancer. It outperforms standard clinicopathologic prognostic criteria and obviates the need to retrieve ≥12 lymph nodes for accurate prognostication. It identifies those patients most likely to develop recurrent disease within 3 years after curative surgery and, thus, those most likely to benefit from adjuvant treatment. Cancer 2012. © 2012 American Cancer Society.

## INTRODUCTION

A paradigm shift in the optimal clinical management of lymph node-negative, invasive colorectal carcinoma (CRC) is being driven by the realization that a significantly higher proportion of these tumors behave like lymph node-positive CRC in terms of recurrence, mortality rates, and response to adjuvant chemotherapy than previously thought. Although most early stage (I-II) CRC is cured by resection alone, 1 in 4 patients eventually develops a recurrence, and the vast majority of recurrences prove fatal.^1^, ^2^ Because the relative incidence of early stage CRC is steadily increasing as a result of enhanced screening efforts,^3^ predicting which of these patients will recur after surgery and benefit most from adjuvant treatment is a significant and growing, unmet medical need in terms of decreasing mortality, improving quality of life, and reducing the cost of care.

Standard prognostic pathologic staging of CRC, eg, the American Joint Committee on Cancer (AJCC) TNM staging system,^2^ provides only an anatomic snapshot of lymph node-negative tumors and lymph node-positive tumors: “lymph node-negative”: stages I and II (pathologic tumor classification T1- T4[pT1-pT4], pathologic lymph node status 0 [pN0], clinically negative for metastasis [cM0]), tumor invades submucosa to direct invasion of other organs or structures (pT1-pT4), no regional lymph node metastasis [pN0], no distant metastasis [cM0]; “lymph node-positive”: stage III (pT1-pT4 pN1-pN2 cM0), the same as stages I and II plus ≥1 regional lymph node metastasis (pN1-pN2). Illustrated by the heterogeneity and overlap of 5-year relative survival rates within and between stages, the system notably fails to adequately account for the underlying molecular and genomic complexity of an individual's specific cancer, information that is key to understanding its natural history and, hence, the true prognosis.

On the basis of TNM staging, current National Comprehensive Cancer Network (NCCN) Clinical Practice Guidelines in Oncology (NCCN Guidelines)^4^ recommend that patients with curatively resected stage I (pT1-pT2 pN0 cM0) colon and rectal cancers can be followed by active surveillance alone, including endoscopically removed malignant pedunculated and sessile polyps with “favorable histologic features” (grade 1-2, no lymphovascular invasion [LVI], and clear margins). However, this guidance does not recognize the 10% to 15% of patients with stage I CRC who will recur and potentially could benefit from adjuvant therapy,^1^, ^2^, ^5^ reflecting the need for better prognostication of these tumors, including the identification of those patients with T1 CRC who can be treated adequately with local excision alone.^6^ For patients with stage II (pT3-pT4 pN0 cM0) colon cancer (CC), all patients also can be followed by active surveillance alone, but it is recommended that adjuvant chemotherapy be considered if 1 or more “high-risk factors for systemic recurrence” are present: T4; grade 3/4; LVI; bowel obstruction; <12 lymph nodes examined; perineural invasion (PNI); localized perforation; or close, indeterminate, or positive resection margins.^4^

Without prognostic stratification, current adjuvant chemotherapeutic regimens appear to improve the survival of patients with stage II CC by no greater than 5%.^4^ Lin et al^7^ recently demonstrated prospectively that, whereas patients who had untreated stage II CC with “high-risk” pathologic features had poorer 3-year disease-free survival (DFS) than those without such features, these “high-risk” patients benefited significantly from 5-fluorouracil–based adjuvant chemotherapy (12% and 14% absolute increase in 3-year DFS and 5-year overall survival, respectively); notably, “low-risk” patients did not benefit. The full degree to which adjuvant chemotherapy can benefit patients with stage I CRC and patients with stage II CC who are deemed “high-risk” for recurrence by more discriminative molecular prognostics is unknown.

The objective of efforts directed at creating clinically useful molecular prognostics is to determine a tumor's inherent aggressiveness, ie, the propensity for early formation of fast-growing, occult micrometastases that are likely to escape detection at the time of initial surgical removal and seed the emergence of a recurrence. Increasingly, this involves the integration and analysis of large amounts of diverse data to produce an accurate, yet inexpensive, prognostic tool. Previously, we successfully used a proprietary version (Evolver) of genetic programming (GP), a machine learning analytic technique based on the mechanisms of natural selection and population dynamics, to generate a molecular signature that defined bladder cancer lymph node status solely by analyzing primary tumor tissue.^8^ We hypothesized that, for the current study, Evolver likewise could produce a clinically useful tumor gene-derived molecular signature that was applicable to all patients with lymph node-negative, invasive CRC and that would outperform current standard clinicopathologic prognostic criteria.

## MATERIALS AND METHODS

### Prognostic Signature Generation and External Validation: Strategic Workflow

**Figure 1:**
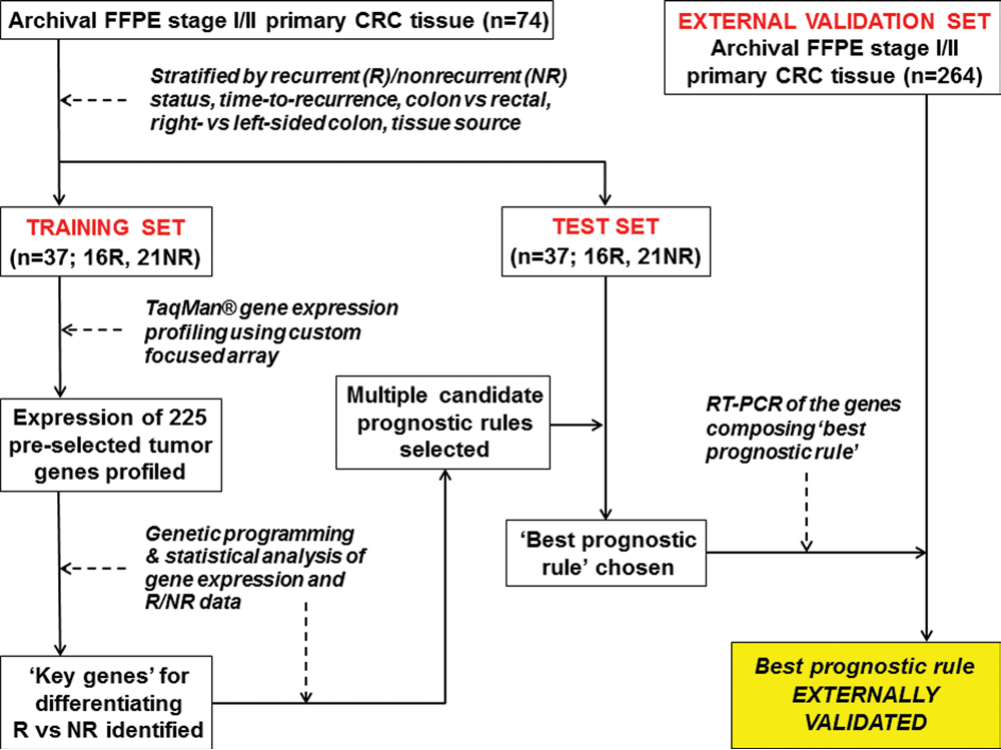
This chart illustrates the strategy for generation and external validation of the molecular prognostic signature. FFPE indicates formalin-fixed, paraffin-embedded; CRC, colorectal carcinoma; R, recurrent; NR, nonrecurrent; RT-PCR, reverse transcriptase polymerase chain reaction.

The strategic workflow for generation and external validation of a molecular prognostic signature that predicts relative risk (“high” vs “low”) for 36-month CRC recurrence (local and/or distant) is illustrated in Figure 1.

### Training and Test Sets

Seventy-four archival, clinically annotated, formalin-fixed, paraffin-embedded (FFPE) primary carcinoma tissues that were obtained at initial surgical resection with curative intent (R0) from 60 patients with CC (pT1-pT4 pN0 cM0) and 14 patients with rectal cancer (pT2-pT4 pN0 cM0) were retrieved from 1 US site (Rochester, Minn; n = 45) and 2 European sites (Moscow, Russian Federation). No patients had received neoadjuvant or adjuvant therapy. The 36-month recurrent (R) and nonrecurrent (NR) status was confirmed for each patient in a review of medical records by site personnel. Informed consent was obtained for all patients. After stratification by recurrence status, time-to-first recurrence, CC versus rectal cancer, right-sided versus left-sided colon, and tissue source, the 74 patients were randomly divided into a Training Set (n = 37; 16 R and 21 NR) and an equally sized Test Set (n = 37; 16 R and 21 NR) (Table 1).

**Table 1:** Patient Clinicopathologic Characteristics

	No. of Patients (%)		External Validation Set^a^	
Characteristics of Evaluable Patients	Training Set	Test Set	R (% of R)	NR (% of NR)
Total no. of patients	37 (100)	37 (100)	92 (100)	172 (100)
Age at surgery: Median [range], y	63 [33-89]	67 [22-90]	64 [23-90]	65 [29-90]
Archival tissue storage: Median [range], y	7 [2-16]	8 [3-15]	7 [3-17]	8 [4-17]
Sex				
Men	16 (43)	17 (46)	35 (38)	81 (47)
Women	21 (57)	20 (54)	57 (62)	91 (53)
Race				
Caucasian	35 (97)	36 (100)	88 (96)	138 (83)
African American	0 (0)	0 (0)	1 (1)	25 (15)
Asian	0 (0)	0 (0)	1 (1)	3 (2)
Other	1 (3)	0 (0)	1 (1)	0 (0)
Tumor classification–Colon cancer				
T1	0 (0)	1 (4)	1 (1)	6 (4)
T2	7 (21)	2 (7)	8 (10)	21 (13)
T3	21 (64)	15 (56)	40 (48)	98 (59)
T4	5 (15)	9 (33)	35 (42)	42 (25)
Anatomic location–Colon cancer				
Right sided	20 (61)	14 (52)	45 (55)	97 (58)
Left sided	13 (39)	13 (48)	37 (45)	69 (42)
Tumor classification–Rectal cancer				
T1	0 (0)	0 (0)	0 (0)	2 (40)
T2	1 (25)	4 (40)	8 (100)	3 (60)
T3	3 (75)	5 (50)	0 (0)	0 (0)
T4	0 (0)	1 (10)	0 (0)	0 (0)
Adenocarcinoma subtype				
Nonmucinous	32 (86)	34 (92)	85 (93)	149 (87)
Mucinous	5 (14)	3 (8)	7 (7)	23 (13)
Histologic grade				
1	4 (11)	4 (11)	12 (13)	30 (18)
2	9 (24)	15 (41)	65 (71)	123 (72)
3	20 (54)	14 (37)	14 (15)	18 (11)
4	4 (11)	4 (11)	0 (0)	0 (0)
No. of lymph nodes examined				
<12	19 (51)	22 (59)	65 (71)	115 (67)
≥12	18 (49)	15 (41)	27 (29)	57 (33)
Median no. [range]	10 [2-45]	9 [3-33]	9 [2-59]	9 [2-59]
Lymphovascular invasion				
Present	7 (30)	15 (58)	41 (47)	40 (24)
Absent	16 (70)	11 (42)	47 (53)	126 (76)
Perineural invasion				
Present	0 (0)	0 (0)	11 (31)	10 (11)
Absent	14 (100)	11 (100)	25 (69)	77 (89)
Bowel obstruction/perforation				
Present	2 (5)	1 (3)	13 (14)	2 (1)
Absent	35 (95)	36 (97)	77 (86)	140 (99)
Recurrence status/location				
Recurrent				
Local	3 (19)	2 (13)	18 (22)	
Distant	11 (69)	14 (88)	63 (78)	
Local and distant	2 (13)	0 (0)	0 (0)	
Nonrecurrent	21 (57)	21 (57)		

Abbreviations NR, did not recur; R, recurred.

a No. of patients; R versus NR designation indicates status at 36 months post-surgery.

### Tissue Processing, RNA Extraction, and cDNA Generation

For each patient in the study, an independent gastrointestinal pathologist circled the location of the highest concentration of CRC cells (routinely, >80%) on a representative hematoxylin and eosin (H&E)-stained tissue slide and verified its histology. Slides with >50% tumor necrosis were disqualified. T-classification and negative surgical margins were confirmed by reviewing gross and microscopic pathology reports. By using the H&E slide as a guide, corresponding unstained tumor tissue affixed to separate glass slides was macrodissected and scraped into RNAse-free microfuge tubes using a disposable scalpel. The tissue was deparaffinized in xylene, and RNA was extracted and purified using the RecoverAll Total Nucleic Acid Isolation Kit (Applied Biosystems/Ambion, Austin, Tex). The purity and quantity of RNA solutions were determined by measuring ultraviolet absorption ratios of 260/280 nm using the Nanodrop 1000 UV/Vis spectrophotometer. A minimum of 100 ng RNA was transcribed into single-stranded cDNA with the High Capacity cDNA Reverse Transcription Kit (Applied Biosystems, Foster City, Calif) using random hexamers as primers.

### Gene Expression Profiling Using TaqMan Low-Density Arrays

Tumor gene expression was assessed by reverse transcriptase-polymerase chain reaction (RT-PCR) using custom 384-well TaqMan Low-Density Arrays (Applied Biosystems). A diverse panel of 417 cancer-associated genes was preselected for the arrays based on existent literature. RT-PCR primers and probes were designed by Applied Biosystems.

After 100 μL of cDNA (1 ng/μL) per 48 wells were applied to the microfluidic cards, all assays were performed in duplicate using the 7900HT Fast Real-Time PCR System (Applied Biosystems). Output data indicated the number of PCR cycles needed to reach a constant threshold set at 0.2 on the amplification curve, ie, the cycle threshold (Ct). The data were normalized using 5 housekeeping (HK) genes (beta-2-microglobulin [*B2M*]; glucuronidase beta [*GUSB*]; polymerase [RNA] II [DNA directed] polypeptide L, 7.6 kDa [*POLR2L*]; proteasome [prosome, macropain] subunit, beta type, 6 [*PSMB6*]; and ubiquitin C [*UBC*]) to correct for potential technical variability and deviation in RNA integrity and quantity in each assay. Each pair of individual gene expression replicates was inspected for congruence, and a correlation coefficient was generated for each. The replicates were averaged, and the resulting data were normalized by subtracting the Ct for each rule gene (RG Ct) from the average of the 5 HK genes (Ave. 5HK Ct). Because Ct values are expressed as logarithmic numbers to the base 2, the data were linearized by taking the antilog, and the result was scaled by a factor of 100 as follows:

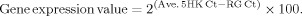
1

Throughout the study, the following minimal criteria for acceptance of extracted RNA and RT-PCR results were used: 1) RNA concentration, ≥10 ng/μL; 2) RNA 260/280 nm ratio, ≥1.8; 3) average expression of the 5 HK genes, ≤32.0 Ct; 4) all individual Ct values, ≤35; and 5) coefficients of determination, r^2^≥0.90.

### Genetic Programming

**Figure 2:**
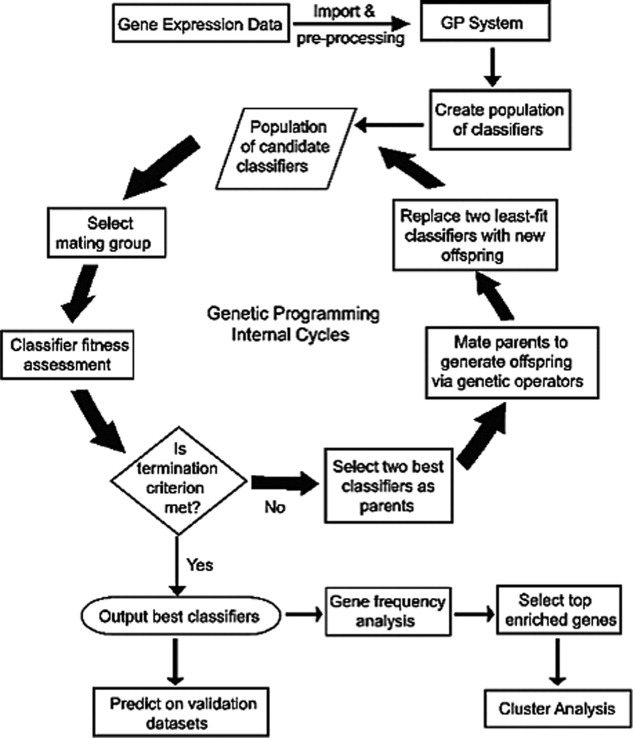
This flow chart illustrates the genetic programming (GP) (Evolver) process. (Reproduced with permission: Yu J, Yu J, Almal AA et al Feature selection and molecular classification of cancer using genetic programming. *Neoplasia*. 2007;9:292-303^9^).

The Evolver GP platform was used in a supervised learning mode on the Training Set (n = 37) to develop dichotomous classifier programs (“rules”) that predicted whether a tumor was R or NR within 36 months after curative surgery, as previously described.^9^ A schematic of the process is presented in Figure 2. The complexity of the rules generated was restricted to prevent overfitting by the strict use of mathematical, logical, and comparison operators, and gene use was restricted to ≤7 genes per rule. The rule produced also was constrained to the following form:

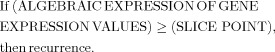
2

The relatively small data set necessitated using N-fold cross-validation to estimate the ability of the classifier to generalize to unseen samples. A reserved Test Set was used to assess the performances of multiple candidate rules selected as among the best generated using the Training Set. Although gene use frequency was influential, the most important measure of fitness was the area under the receiver operating characteristics curve. The “best prognostic rule,” containing a subset of the key differentiating genes, was selected based on comparison of performance characteristics of all candidate rules tested. Finally, the Training and Test Sets were combined, and the slice point for the final rule was refined using this larger set.

### External Validation Set

To be included in the External Validation Set (EV Set), each potential case needed to pass 2 successive eligibility screens. First, the associated clinicopathologic information needed to satisfy the major eligibility criteria detailed in Table 2, ensuring that the patient underwent potentially curative surgery for lymph node-negative, invasive CRC and that 36-month R or NR status could be confirmed. Inquiries by site personnel regarding the presence/absence of each of the “high-risk factors for systemic recurrence” per NCCN Guidelines were reviewed for internal consistency with pathology reports and other data available for each patient. Second, after passing the initial screening, each case needed to satisfy the aforementioned minimal criteria for acceptance of extracted RNA and RT-PCR results. From a pool of 484 patients with lymph node-negative CRC who satisfied the initial clinicopathologic screening, 264 (55%) subsequently passed the RNA/RT-PCR screening step and were included in the final EV Set. The predominant reasons for rejection of a patient during the second screening step were: 1) the average expression of the 5 HK genes exceeded 32.0 Ct (128 of 220 patients; 58%), 2) no amplification of ≥1 requisite gene (61 of 220 patients; 28%), and 3) insufficient amount or quality of RNA (18 of 220 patients; 8%).

**Table 2:** Major Tissue and Clinical Data Eligibility Criteria for the External Validation Set

Inclusion criteria
• Confirmed single, primary AJCC stage I-II colon carcinoma (pT1-pT4 pN0 cM0) or stage I rectal (including rectosigmoid) carcinoma (pT1-pT2 pN0 cM0)
• Confirmed negative surgical margins (>1 mm) of resection (R0): radial, distal, proximal
• Confirmation that, after surgery, the patient either
1) Recurred within 3-36 mo (includes CRC-related death) OR
2) Survived for 36 mo without recurrence (confirmation of continual “no evidence of disease” at/after 36 mo post surgery required)
Exclusion criteria
• Neoadjuvant or adjuvant radiotherapy or chemotherapy
• Metachronous CRC tumors evident during the initial 36 mo of follow-up, unless local/distant CRC recurrence of the index lesion had already been documented

Abbreviations: AJCC, American Joint Committee on Cancer; CRC, colorectal carcinoma; cM, clinical metastatic level; pN, pathologic lymph node status; pT, pathologic tumor classification.

The final EV Set was comprised of archival, FFPE, primary CRC adenocarcinoma tissues (median storage time, 7 years; range, 3-17 years) that were obtained at initial curative resection from patients at 15 hospitals in 4 countries (United States, Russian Federation, Pakistan, and Germany). These sources were different from those used in the Training/Test Sets. For all cases, appropriate patient informed consent had been obtained. The clinicopathologic characteristics of the final EV Set are detailed in Table 1. A subset (n = 251) of patients in the EV Set comprised of only patients with stage I and II CC (the EV Set-CC) also was used to assess the performance characteristics of the final prognostic rule.

### Comparison With National Comprehensive Cancer Network Guidelines

A prospective review of the EV Set indicated that sufficient clinicopathologic information had been obtained for 241 of the 264 EV Set patients (91%) to compare the prognostic abilities of a molecular signature and the NCCN Guidelines for CC (version 1.2012) and rectal cancer (version 1.2012). Whereas all patients with stage I CRC were included, because NCCN Guidelines recommend active surveillance alone for these patients, the presence or complete absence of the NCCN Guidelines' “high-risk factors for systemic recurrence” were required to qualify patients with stage II CC. For the 23 patients with stage II CC who were excluded from this analysis, the prevalence of missing data was as follows: 1) PNI (16 of 23 patients; 70%), 2) bowel obstruction/perforation (8 of 23 patients; 35%), and 3) LVI (3 of 23 patients; 13%).

### Statistical Analysis

Sample size calculations were based on the minimum number of patients required to provide 80% power (alpha = .05; assuming equal proportions of predicted high-risk and low-risk patients) to detect a difference in 3-year recurrence-free survival between low-risk patients versus high-risk patients of 18 percentage points (43% vs 25%). A statistically significant difference between these 2 patient groups would demonstrate the test's potential prognostic value and clinical utility. Exact binomial 95% confidence intervals (CIs) were calculated for proportions. Survival analysis *P* values comparing recurrence-free survival between high-risk and low-risk patients were calculated using the log-rank test, and hazard ratios were derived from Cox proportional hazards regression. Sample size calculations were done using PASS 2005 Power Analysis and Sample Size software (NCSS, Kaysville, Utah), and statistical analyses were conducted using SAS statistical software (SAS version 9.2; SAS Institute, Inc., Cary, NC) and MedCalc (version 11.6.1; MediCalc Software, Mariakerke, Belgium).

Sensitivity and specificity were compared between the molecular prognostic test and the NCCN Guidelines among the 241 patients with CRC who had sufficient data for such comparisons. Analyses were performed only among patients for whom the test results differed using an exact McNemar test. A comparison of sensitivities was made by comparing the relative proportions of patients with high-risk test results among patients who had a recurrence within 3 years, and a comparison of specificities was made by comparing the relative proportions of patients with low-risk test results among patients who did not have a recurrence within 3 years. A comparison of hazard ratios was done by including both test results in a Cox proportional hazards regression model and comparing their parameter estimates.

## RESULTS

### Generation of Rules

After applying the above-described minimal criteria for acceptance of RT-PCR data for the 417 preselected cancer-associated genes, 192 genes were eliminated from consideration, which left a subset of 225 select genes that was used in subsequent analyses. While varying the maximum number of genes allowed in each rule, 4 separate sets of runs were made totaling 4800 separate runs (4 folds × 300 runs × 4 sets), using the Training Set. By using statistical analysis of the selection of genes preferred by the 4800 candidate rules produced, 18 “key genes” were selected that consistently yielded the highest performing results using the Test Set (Table 3).

**Table 3:** Genes Used to Generate Final Candidate Rules

Gene Symbol	Full Name	TaqMan Probe Length, No. of Nucleotides^a^	TaqMan Gene Expression Assay^a^	Frequency of Gene Use in Candidate Rules, %
“Key genes” (n = 18)				
*MMP2*	Matrix metallopepidase 2	84	Hs01548733_m1	36
*AKT1*	v-Akt murine thymoma viral oncogene homolog 1	66	Hs00178289_m1	23
*RPS10*^b^	Ribosomal protein S10	108	Hs01652367_gH	19
*NFKB1*	Nuclear factor of kappa light polypeptide gene enhancer in B-cells 1	73	Hs00231653_m1	16
*CD82*	Cluster of differentiation 82 molecule	86	Hs00356310_m1	13
*ARHGDIB*	Rho GDP dissociation inhibitor beta	81	Hs00171288_m1	12
*H3F3B*^b^	H3 histone, family 3B	83	Hs00855159_g1	12
*BMI1*^b^	BMI-1 polycomb ring finger oncogene	105	Hs00180411_m1	11
*VEGFA*^b^	Vascular endothelial growth factor A	59	Hs00900055_m1	10
*ETV6*^b^	Ets variant 6	75	Hs01045742_m1	9
*HMOX1*	Heme oxygenase (decycling) 1	82	Hs01110250_m1	9
*ARAF*	v-Raf murine sarcoma 3611 viral oncogene homolog	74	Hs00176427_m1	6
*PTK2*	PTK2 protein tyrosine kinase 2	68	Hs00178587_m1	5
*DIABLO*	Diablo, inhibitor of apoptosis-binding mitochondrial protein	70	Hs00219876_m1	4
*MAX*	MYC-associated factor X	61	Hs00231142_m1	2
*FGFR4*	Fibroblast growth factor receptor 4	74	Hs00242558_m1	1
*ITGB1*	Integrin, beta 1	86	Hs01127543_m1	<1
*MAPK14*	Mitogen-activated protein kinase 14	91	Hs01051152_m1	<1
Housekeeping genes (n = 5)				
*B2M*	Beta-2-microglobulin	64	Hs00187842_m1	
*GUSB*	Glucuronidase, beta	81	Hs99999908_m1	
*POLR2L*	Polymerase (RNA) II (DNA directed) polypeptide L, 7.6 kDa	74	Hs00360764_m1	
*PSMB6*	Proteasome (prosome, macropain) subunit, beta type, 6	93	Hs00382586_m1	
*UBC*	Ubiquitin C	71	Hs00824723_m1	

a TaqMan products were obtained from Applied Biosystems (Foster City, Calif).

b This gene is a component of the 5-gene “best prognostic rule.”

The “best prognostic rule” selected was an algebraic expression of the gene expression profiles of BMI-1 polycomb ring finger oncogene (*BMI1*); ets variant 6 (*ETV6*); H3 histone, family 3B (*H3F3B*); ribosomal protein S10 (*RPS10*); and vascular endothelial growth factor A (*VEGFA*). This dichotomous 5-gene signature subsequently underwent external validation:

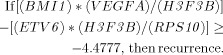
1

Although the slice point chosen separated a range of values (−92.8245 to 69.7508) in the Training and Test Sets into 2 groups (<−4.4777 and ≥−4.4777), lower or higher values within each of the 2 groups did not relate to any gradation of recurrence risk.

### External Validation of the 5-Gene Prognostic Signature

RT-PCR-generated expression profiles of the 5 cancer-associated genes comprising the molecular prognostic were assembled by an individual who was blinded to the corresponding clinical recurrence status. The designations “low-risk” and “high-risk” for recurrence were then assigned programmatically according to the prognostic rule algorithm. A separate individual who was blinded to the corresponding gene expression profiles compared the computer-generated recurrence designations with the actual patient outcome data.

**Figure 3:**
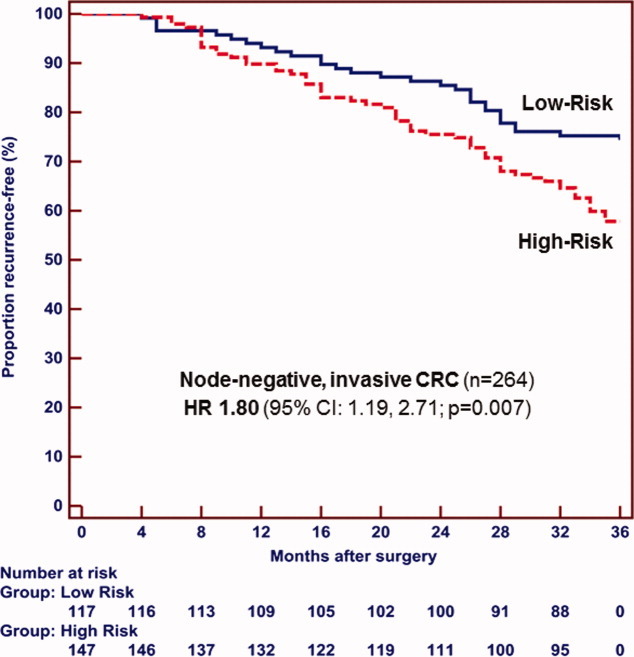
This chart illustrates the ability of the 5-gene molecular signature to differentiate lymph node-negative, invasive colorectal cancer (CRC) tumors in the external validation set (n = 264) for patients at “low risk” versus patients at “high risk” of developing a recurrence within 36 months after surgery. CI indicates confidence interval; HR indicates hazard ratio.

By using the full EV Set (n = 264), the molecular signature correctly classified 62 of 92 R cases and 87 of 172 NR cases (sensitivity, 0.67 [95% CI, 0.57-0.77]; specificity, 0.51 [95% CI, 0.43-0.58]). In the CRC population that we studied, “high-risk” patients had a significantly greater probability of recurrence by 36 months (42%) than “low-risk” patients (26%; positive predictive value [PPV], 0.42 [95% CI, 0.34-0.51]; negative predictive value [NPV], 0.74 [95% CI, 0.65-0.82]; hazard ratio, 1.80 [95% CI, 1.19-2.71]; *P* = .007; log-rank test) (Fig. 3)

Univariate results for each baseline variable from a Cox proportional hazards regression model that included each baseline variable plus the dichotomous 5-gene prognostic test result are shown in Table 4. The analysis demonstrated that, among the variables tested, the 5-gene signature performed independent of histologic grade, T-classification, number of regional lymph nodes examined, LVI, bowel obstruction/perforation, anatomic tumor location, age at surgery, sex, race, and adenocarcinoma subtype.

**Table 4:** The 5-Gene Molecular Signature: Univariate Predictors

		Variable		5-Gene Molecular Signature	
Baseline Variable	No. of Patients	HR	*P*	Adjusted HR	*P*
Race (Caucasian vs non-Caucasian; effect of Caucasian)	257	4.063	.018	1.607	.035
Bowel obstruction/perforation (positive vs negative)	232	4.016	<.001	1.656	.027
Perineural invasion (positive vs negative)	123	2.432	.021	1.377	.405
Lymphovascular invasion (positive vs negative)	254	2.023	.001	1.848	.010
Adenocarcinoma subtype (mucinous vs nonmucinous; effect of nonmucinous)	264	1.749	.156	1.839	.006
Histologic grade (continuous)	262	1.354	.131	1.859	.006
Sex (man/woman; effect of woman)	264	1.315	.203	1.772	.010
Right-sided vs left-sided tumor (effect of left-sided tumor)	261	1.202	.385	1.695	.019
T-classification (continuous)	264	1.106	.511	1.720	.019
No. lymph nodes examined (continuous)	264	0.997	.836	1.786	.010
Age at surgery (continuous)	264	0.994	.534	1.772	.011

Abbreviations: HR, hazard ratio.

On the basis of the univariate results, a backward-elimination, stepwise Cox proportional hazards regression was performed with all baseline variables plus the 5-gene prognostic test result. At each step, the variable with the highest *P* value was eliminated (except the molecular test, which was forced to remain in the model) until all baseline variables had a *P* value < .05. The multivariate analysis (Table 5) demonstrated that, among all non-test variables examined (PNI was not included, because only 91 of 264 patients had data available on PNI and all other variables), only LVI and bowel obstruction/perforation remained independent. When using only the subset of patients in which the status of LVI and clinical bowel obstruction/perforation were both known (n = 229), the adjusted hazard ratio for the molecular test (1.64; 95% CI, 1.04-2.61) remained statistically significant and independent of the other 2 variables (*P* = .035). By using the EV Set-CC (n = 251), the molecular signature correctly classified 55 of 84 patients in the R group and 83 of 167 patients in the NR group (sensitivity, 0.65; specificity, 0.50; PPV, 0.40; NPV, 0.74; hazard ratio, 1.63; 95% CI, 1.06-2.50; *P* = .031).

**Table 5:** The 5-Gene Molecular Signature: Multivariate Predictors (N = 229)

Variable^a^	HR (95% CI)	*P*
Bowel obstruction/perforation (positive vs negative)	4.118 (2.270-7.471)	<.001
Lymphovascular invasion (positive vs negative)	1.766 (1.156-2.697)	.009
Five-gene molecular signature (high-risk vs low-risk for 36-mo recurrence)	1.644 (1.035-2.610)	.035

Abbreviations: CI, confidence interval; HR, hazard ratio.

a Perineural invasion was not included in the model because of the amount of missing data.

### Comparison With National Comprehensive Cancer Network Guidelines

Differences in sensitivity and specificity were compared among the 241 evaluable patients in the EV Set who had sufficient information for analysis. Among the 90 patients with CRC who developed a recurrence within 3 years, the proportions with high-risk test results were 69% (62 of 90 patients) using the molecular prognostic test and 73% (66 of 90 patients) using NCCN Guidelines (*P* > .10). Among the 151 patients with CRC who did not develop a recurrence within 3 years, the proportions with low-risk test results were 48% (73 of 151 patients) using the molecular prognostic test and 26% (39 of 151 patients) using NCCN Guidelines (*P* < .001). Of the 112 false-positive designations made using NCCN Guidelines, insufficient (<12) lymph node sampling (93 of 112 patients; 83%) and T4 categorization (42 of 112 patients; 38%) contributed to the vast majority. Among the 241 evaluable patients, the hazard ratios for the 5-gene prognostic signature and NCCN Guidelines were 1.76 (95% CI, 1.13-2.75; *P* = .013) and 0.897 (95% CI, 0.56-1.43; *P* = .648), respectively. When the 2 tests were included in the same model and adjusted for each other, they differed significantly (*P* = .021).

## DISCUSSION

The external validation results presented in this report confirm that at least 2 distinct molecular prognostic subgroups exist within the histopathologically defined, lymph node-negative, invasive CRC population and that determining the subgroup to which a particular tumor belongs using the 5-gene signature (OncoDefender-CRC) outperforms current NCCN Guidelines. The ability of this simple assay to differentiate patients at high risk versus low risk for tumor recurrence within 3 years after potentially curative surgery was validated not only for lymph node-negative CRC but also for lymph node-negative CC alone. OncoDefender-CRC should prove useful to: 1) the medical oncologist when deciding which patients with CRC are most appropriate for adjuvant treatment; 2) the gastroenterologist and surgeon when formulating the optimal follow-up strategy for a patient undergoing colorectal polypectomy and presenting with a malignant polyp without accompanying lymph node data; and 3) the investigator who needs to stratify early stage CRC patient populations for testing of novel adjuvant therapies. It is anticipated that, when this test is used prospectively and FFPE tissue storage times are reduced and handling procedures optimized, far fewer tumors will fail quality screening than failed in this retrospective study.

The 18 “key genes” that were used most commonly in the candidate rules exert stimulatory, inhibitory, and/or regulatory effects in 7 broad pathways^8^ that commonly are dysregulated in cancer (available at: http://www.ncbi.nlm.nih.gov/gene; [accessed October 10, 2011]) and were as follows: signal transduction (*AKT1*, *ARAF*, *CD82*, *ITGB1*, *MAPK14*, *PTK2*), gene regulation (*ETV6*, *H3F3B*, *MAX,NFKB1*, *BMI1*), invasion (*ARHGDIB*, *MMP2*), cell growth regulation (*FGFR4*, *RPS10*), angiogenesis (*VEGFA),* apoptosis (*DIABLO*), and antioxidation (*HMOX1*). Examination of the algebraic 5-gene signature reveals that higher expression levels of *BMI*, *VEGFA*, and *RPS10* relative to *ETV6* and *H3F3B* expression maximize the chances that “recurrence” is predicted. Consistent with this, it is known that *BMI1* represses growth arrest and apoptosis and that *VEGFA* can induce angiogenesis and cell migration (available at: http://www.ncbi.nlm.nih.gov/gene; [accessed October 10, 2011]). Although the effects of varying expression levels of *RPS10* (encodes a ribosomal protein), *ETV6* (encodes a transcription factor), and *H3F3B* (encodes a histone protein) on the malignant phenotype are less well defined, their presence in the 5-gene rule are not inappropriate for a molecular signature of a tumor's inherent aggressiveness.

The 5-gene molecular assay performs better than nearly all recognized clinicopathologic prognostic factors for early stage CRC, most notably T-classification, histologic grade, and the number of regional lymph nodes examined. Obviating the need to collect and examine a minimum of 12 regional lymph nodes for metastases is a practical advantage for using the molecular test, especially when insufficient numbers of lymph nodes are obtained during surgery or when no lymph nodes are available at all, eg, after endoscopic polypectomy. It is noteworthy that, when the NCCN Guidelines were used to assess the risk of tumor recurrence in this study population, the bulk of the false-positive categorizations made, ie, designating relatively indolent disease (“low-risk”) as aggressive (“high-risk”), resulted from sampling <12 lymph nodes and being unduly influenced by T4 status. The finding that the gene signature outperformed T-classification and histologic grade strongly supports the hypothesis that molecular characterization transcends traditional visual pathologic staging in defining the biology underlying early stage CRC recurrence.

Although the concomitant knowledge of the status of bowel obstruction/perforation and LVI remained an independent prognostic variable in the multivariate analysis, such knowledge often is not available and, when it is, may not be reliable for prognostic assessment. Although bowel perforation rarely occurs in lymph node-negative CRC (<5%),^10^ when it does, the uniformity of its prognostic significance has been questioned.^11^ Likewise, depending on the definition of “bowel obstruction” used (mechanical vs functional; partial vs complete), the reported incidences of preoperative bowel obstruction range from <10% to >40%.^12^, ^13^ Regarding LVI, Harris et al^14^ reported that interobserver agreement in its diagnosis in stage II CRC is only slightly higher than would be produced by chance alone (kappa = 0.24). Taken together, these observations suggest that bowel obstruction/perforation and LVI are considerably ill-defined, subjective, and/or rarely observed variables in patients with early stage CRC and, as such, should not be regarded as significant detractors from the clinical utility of the 5-gene test for individual patients. The finding that the NCCN Guidelines, which involve the assessment of bowel obstruction/perforation and LVI, were significantly inferior in prognostic performance to the performance of the molecular signature supports this assertion.

PNI, which occurs in 10% to 15% of patients with lymph node-negative CRCs, has only recently been recognized as an independent prognostic factor in this disease.^15^ Although our univariate analysis indicated that PNI is a significant prognosticator in the current study population, further investigation is needed before conclusions can be reached regarding the relative prognostic abilities of PNI and the 5-gene molecular assay in a multivariate analysis.

Microsatellite instability, which is characterized by the inactivation of selected mismatch repair genes, is observed in 15% of patients with CRC and is a recognized prognostic factor in patients with stage II CC, but, to date, not in stage I CC.^16^ Because microsatellite instability/mismatch repair data were not available for the EV Set, it was not included in the univariate and multivariate analyses that were conducted. However, even if future studies involving OncoDefender-CRC were to indicate that microsatellite instability/mismatch repair was an independent prognosticator for patients with stage II CC, it would affect no more than 8% to 10% of the early stage CRC population applicable to the test.

The only marketed, primary tumor-specific molecular prognostic for early stage CRC is the RT-PCR–based, continuous “Oncotype DX Colon Cancer Assay” score (Genomic Health, Inc., Redwood City, Calif), which is specific for T3 CC with proficient mismatch repair genes.^17^ Compared with the ability of the Oncotype DX assay to correctly identify patients with lymph node-negative CC who are at high risk for recurrence within 3 years after curative surgery alone and to differentiate them from those at low risk for such recurrence, the dichotomous OncoDefender-CRC has greater sensitivity (0.65 [55 of 84 patients] vs 0.52 [37 of 71 patients]) and a higher hazard ratio (1.63 [*P* = .031] vs 1.47 [*P* = .046]). Although, in multivariate analysis, T-classification (*P* = .004), anatomic tumor location (*P* = .032), and age (*P* = .034) remain independent predictors of 3-year recurrence when using the Oncotype DX assay, OncoDefender-CRC outperforms these prognostic variables while also eliminating the need for an “intermediate-risk” group that, in 30% of patients, complicates the interpretation of the Oncotype DX assay results. To our knowledge, there are no published reports describing how the prognostic ability of the Oncotype DX Colon Cancer Assay compares with the prognostic ability of PNI, bowel obstruction, or localized perforation in CC.

Most notably, OncoDefender-CRC is the only molecular CRC prognostic assay that has been shown to outperform a complete set of standard clinicopathologic prognostic criteria: specifically, the current NCCN Guidelines. It affords clinicians who need guidance in postoperative clinical management with an improved prognostic tool that is applicable to all stages of lymph node-negative, invasive CRC.

In conclusion, a 5-gene prognostic signature (OncoDefender-CRC) capable of differentiating between patients with lymph node-negative, invasive CRCs at “high risk” from those at “low risk” for recurrence within 3 years after curative resection was generated using GP analysis of tumor gene expression profiles of archival FFPE tumor tissue. To our knowledge, this is the first molecular prognostic test to be validated in both stage I CRC and stage II CC. It outperforms standard clinicopathologic prognostic criteria and obviates the need to retrieve ≥12 lymph nodes for accurate prognostication. It identifies those patients most likely to recur after curative surgery and, thus, those most likely to benefit from adjuvant treatment.

## FUNDING SOURCES

This study was supported by Everist Genomics, Inc.

**CONFLICT OF INTEREST DISCLOSURES**

Peter Lenehan, Brigitte Gerard, Arpit Almal, and William Worzel are employees of Everist Genomics, Inc.; Eugene Heyman is a paid biostatistical consultant for Everist Genomics, Inc.; and David Fry and Jeanne Ohrnberger are former employees of Everist Genomics, Inc.
